# From preneoplastic lesion to heterogenous tumor: recent insights into hepatoblastoma biology and therapeutic opportunities

**DOI:** 10.1186/s12943-025-02405-8

**Published:** 2025-07-19

**Authors:** Jun Yang, Andrew M. Davidoff, Andrew J. Murphy

**Affiliations:** 1 Department of Surgery, St. Jude Children’s Research Hospital, MS 332, 262 Danny Thomas Place, Memphis, TN 38105 USA; 2 Department of Pathology and Laboratory Medicine, College of Medicine, The University of Tennessee Health Science Center, Memphis, TN 38163 USA; 3 College of Graduate Health Sciences, University of Tennessee Health Science Center, Memphis, TN 38163 USA

## Abstract

Hepatoblastoma is the most common pediatric liver cancer, with the fastest rising incidence among childhood malignancies. Early genomic studies revealed that hepatoblastoma has the lowest mutational burden of any human cancer, however, recent advances in single-cell RNA-seq, multiomics, spatial transcriptomics, and functional genomics screenings have revealed substantial complexity. Diverse cellular subpopulations, divergent WNT signaling, key developmental pathways, and intricate interactions between the tumor cells and tumor immune microenvironment (TME) collectively shape tumor heterogeneity, disease progression, therapeutic responses, and genetic dependencies. Mosaic embryonic loss of heterozygosity (LOH) at chromosome 11p15.5 may be a hepatoblastoma-initiating event, as clonal expansion of 11p15.5 LOH occurs in adjacent normal liver tissue. A cholangiocyte-like subpopulation expresses *FGF19*, in a *SOX4-*dependent, paracrine manner, to drive the proliferation of neighboring embryonal hepatoblastoma cells. WNT-signaling dependent MDK promotes the immunosuppressive TME, which impairs immune cell infiltration. The TME may also be driven by islands of erythroblasts, which influence treatment resistance. Plasticity driven by changes in chromatin accessibility enables differentiation transition between hepatocytic and liver progenitor cell types, which is associated with treatment resistance. Here, we review recent findings in pediatric hepatoblastoma cells, tumor-associated cell types, and genetic dependencies that will serve to advance hepatoblastoma therapy.

**Clinical trial number**: Not applicable.

**Supplementary Information:**

The online version contains supplementary material available at 10.1186/s12943-025-02405-8.

## Introduction

Hepatoblastoma is the most common primary liver cancer in children [1]accounting for about 80% of pediatric liver malignancies, with a peak occurrence in children younger than 3 years. The worldwide incidence of hepatoblastoma is rising faster than any other pediatric cancer [2]. Although surgical resection combined with chemotherapy can cure 95% of patients with low-risk disease and 89% of those with intermediate-risk hepatoblastoma [3]it comes at the cost of adverse effects that have long-term sequelae, including secondary cancer, in young children [4]. For those children with high-risk hepatoblastoma, there is persistent unmet need for new therapies; the probability of 3-year overall survival and event-free survival of high-risk disease are just 52% and 44%, respectively [5].

Hepatoblastoma is an embryonal neoplasm that arises from hepatoblasts [6, 7]the common progenitor of hepatocytes and cholangiocytes. Hepatoblastoma has the fewest somatic mutations among all human cancers [8]suggesting that during the early stages of liver development, hepatic precursor cells are particularly susceptible to simple genetic events that cause oncogenic transformation. Early studies identified genetic mutations in *CTNNB1*, *APC*, *AXIN1*, *AXIN2* in hepatoblastoma [9–15]. Following DNA sequencing studies further validated that mutations in the WNT–β-catenin pathway genes are the main genetic events in hepatoblastoma and also identified other rare genetic mutations in *NFE2L2* and *TERT* genes [16–25]. Despite its relatively simple genomic landscape, hepatoblastoma presents as a biologically and clinically heterogeneous disease, characterized by diverse histologic subtypes and variable clinical manifestations and therapeutic responses. Recent single-cell RNA-sequencing (scRNA-seq), multi-omics, and spatial transcriptomics studies have provided new insights into the cellular origin and tumor heterogeneity of hepatoblastoma and its tumor microenvironment (TME), which may provide opportunities to develop more effective therapies and improve survival.

### From preneoplastic lesion to hepatoblastoma

Genomic sequencing analyses revealed that the vast majority (~ 70–90%) of hepatoblastomas harbor genetic alterations in *CTNNB1*^16–25^, the gene that encodes β-catenin and plays a key role in the WNT-signaling pathway. The second-most frequently affected genomic region in hepatoblastoma is the 11p15.5 locus, with genomic and/or epigenetic aberrations detected in as many as 84% of cases [7, 22, 23].

The high co-occurrence of *CTNNB1* and 11p15.5 alterations in hepatoblastoma suggests that both genetic events are required for tumorigenesis. However, the sequence of genetic events during hepatoblastoma development was not fully understood until recent studies revealed alterations of 11p15.5 in preneoplastic liver cells [22, 26].

**Fig. 1:**
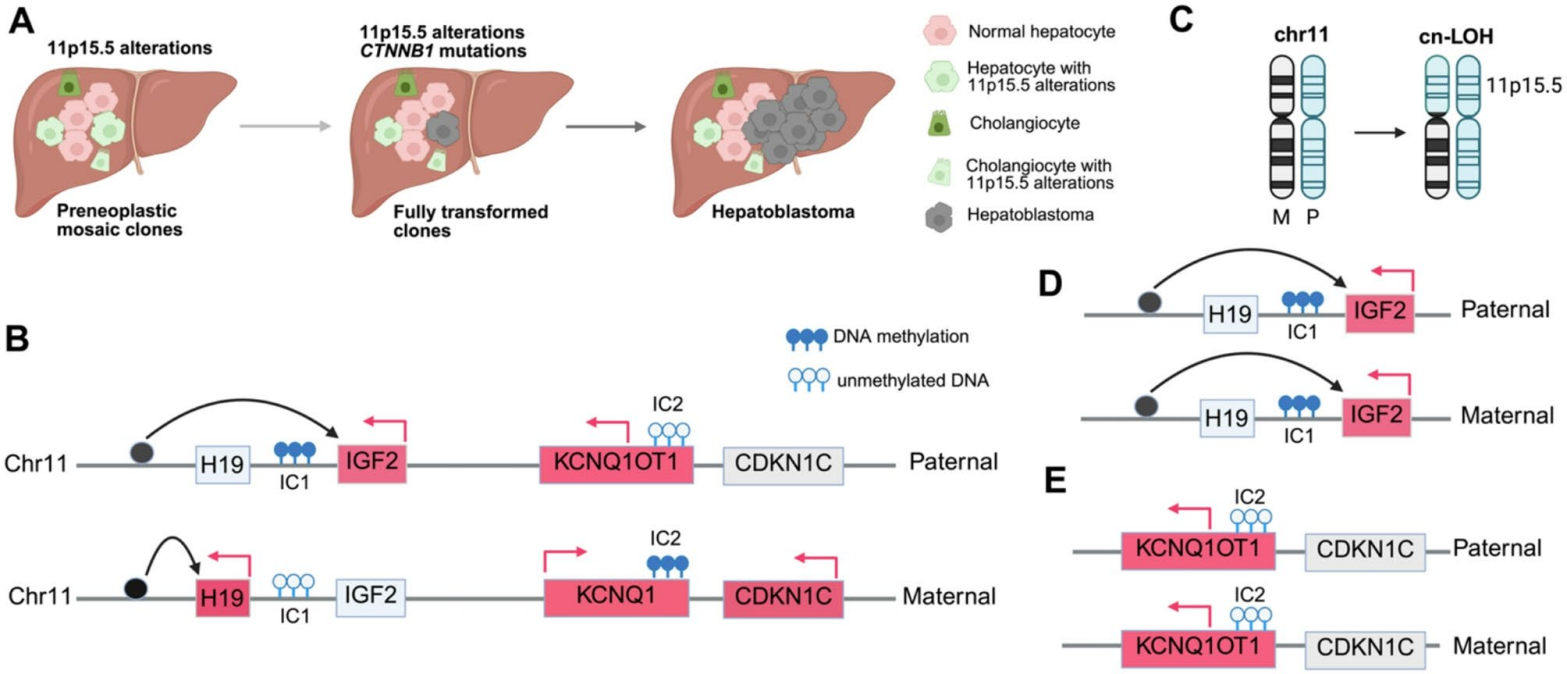
Mosaicism of the 11p15.5 locus precedes the gain-of-function mutations of ***CTNNB1*** during hepatoblastoma tumorigenesis. (**A**) Alterations at the 11p15.5 locus occur in hepatobiliary progenitor cells during embryogenesis, establishing a pre-neoplastic state. This is frequently followed by a second genetic event, most commonly activating mutations in *CTNNB1*, which encodes β-catenin. Together, these molecular events drive the full transformation of hepatocytes, typically resulting in tumor development within the first 3 years of life. (**B**) Epigenetic regulation of the 11p15.5 locus by DNA methylation at the imprinting control 1 (IC1) and 2 (IC2) regions. IC1 is methylated on the paternal allele and unmethylated on the maternal allele, enabling paternal-specific expression of *IGF2* and maternal-specific expression of *H19*. Conversely, IC2 is methylated on the maternal allele and unmethylated on the paternal allele, permitting maternal-specific expression of *CDKN1C*. (**C**) Copy-neutral loss of heterozygosity (cn-LOH, paternal uniparental disomy) at the 11p15.5 locus frequently occurs in a mosaic pattern in hepatocytes, contributing to aberrant gene dosage and epigenetic dysregulation of oncogene *IGF2* and tumor suppressor *CDKN1C*. chr, chromosome; M, maternal; P, paternal. (**D**) IC1 epimutation leads to *IGF2* expression on both alleles. (**E**) IC2 epimutation leads to the silencing of *CDKN1C* on both alleles

Hepatoblastoma is associated with genetic syndromes, such as familial adenomatous polyposis and Beckwith-Wiedemann syndrome (BWS). Germline mutations in the *APC* gene increases hepatoblastoma risk via dysregulated WNT/β-catenin signaling in patients with familial adenomatous polyposis, and 11p15.5-imprinting defects or paternal uniparental disomy causes BWS. However, not all patients with BWS are predisposed to liver cancers, suggesting that alterations in 11p15.5 alone are insufficient to drive tumorigenesis, and a second genetic event is needed. Pilet et al. [26] recently showed that in the nonmalignant liver tissues of some patients with hepatoblastoma but not BWS, 11p15.5 alterations are detected in hepatocytes and cholangiocytes prior to *CTNNB1* mutations being detected. These findings indicate that 11p15.5 alterations occur during the hepatobiliary progenitor stage of embryogenesis, thereby predisposing hepatocytes to transformation by *CTNNB1* mutations (Fig. 1A). Interestingly, we and others have also found 11p15.5 mosaicism in normal kidneys of patients with bilateral Wilms tumors, suggesting that mosaic alteration occurs before the diversion of left and right kidney primordia [27, 28]which may serve as the first genetic hit of cellular transformation during nephrogenesis.

Why are alterations at 11p15.5 important to tumorigenesis for these embryonic malignancies? The 11p15.5 locus is a parentally imprinted region that harbors the *IGF2* oncogene, *H19* noncoding RNA, and the *CDKN1C* tumor suppressor, whose expression is tightly controlled by two imprinting control (IC) regions, IC1 and IC2, that are subject to allele-specific DNA methylation (Fig. 1B). *IGF2* and *H19* share common enhancers, and their expression is reciprocally regulated via IC1 methylation. IC1 is typically methylated on the paternal allele, which prevents CTCF binding [29]thereby allowing enhancers to drive *IGF2* expression. However, IC1 is unmethylated on the maternal allele, which enables the enhancers to drive *H19* expression. Conversely, on the paternal allele, IC2 is unmethylated, which permits the expression of the antisense noncoding RNA *KCNQ1OT1*, which in turn, epigenetically silences *CDKN1C* and *KCNQ1*. The maternal allele is methylated at IC2, thereby allowing *CDKN1C* and *KCNQ1* to be expressed. Abnormal methylation at IC1 or IC2 or chromosomal aberrations like copy-neutral loss of heterozygosity (cn-LOH) at 11p15.5 can lead to dysregulated expression of *IGF2* and *CDKN1C* (Fig. 1C). Abnormal expression of *IGF2*, *H19*, and *CDKN1C* in hepatoblastoma, driven by imprinting defects and/or loss of heterozygosity at 11p15.5, was first observed nearly three decades ago [30–32]. The alterations of 11p15.5 were also found in BWS and other cancers such as Wilms tumor [33, 34]. However, the underlying mechanisms of gene dysregulation in 11p15.5 appear to differ in hepatoblastoma from Wilms tumor and BWS. The 11p15.5 alterations in premalignant liver and kidney tissues display distinct patterns. The cn-LOH predominates in 69% of preneoplastic mosaic livers [26]; IC1 hypermethylation predominates in 58% of premalignant mosaic kidney [27, 28] (Fig. 1D), and IC2 epimutation (loss of methylation) is the major alteration in 64% of BWS cases [35] (**Fig. 1E**). A spatial transcriptomics study revealed that the nontumor liver tissues with mosaic 11p15.5 overexpress *IGF2* and downregulate *H19*, with an alteration of the liver zonation [26]. Additionally, mosaic livers were enriched in extracellular matrix and angiogenesis gene markers, suggesting that 11p15.5 alterations create a pro-angiogenic microenvironment for tumorigenesis.

A multi-omics study by Nirgude et al. [36] showed that livers from patients with BWS exhibit metabolic dysfunction and enrichment of peroxisome proliferator–activated receptor alpha signaling in hepatocytes with 11p15.5 alterations, which is consistent with high expression of lipid metabolism genes in the 11p15.5 mosaic livers of patients with hepatoblastoma [26]. Although the 11p15.5 alterations in premalignant hepatocytes represent an early step in hepatoblastoma development, they can occur late or synchronously with *CTNNB1* alterations [26]. Genetic mouse models have shown that *CTNNB1* mutations alone are insufficient to initiate hepatoblastoma [37, 38]. As a key fetal growth factor, IGF2 may cooperate with β-catenin or other oncogenes to drive tumorigenesis. Our recently developed mouse model of MYC-driven hepatoblastoma showed that tumors express high levels of *Igf2* (ranked #1 among the differentially expressed genes), supporting the notion that IGF2 plays an important role in tumorigenesis [39]. However, the precise molecular mechanisms underlying the cooperation of IGF2 and β-catenin or other oncoproteins remain poorly understood.

Another question that remains unanswered is whether alterations at the 11p15.5 locus are a prerequisite for the development of other cancer types. Notably, analysis of *IGF2* expression across more than 1,000 cancer cell lines revealed that *IGF2* is selectively and highly expressed in embryonal tumors, including Wilms tumor, hepatoblastoma, rhabdomyosarcoma, CIC–DUX4 sarcoma, and synovial sarcoma (Supplementary Fig. 1). This unique pattern of *IGF2* expression suggests a shared oncogenic mechanism in which 11p15.5 alteration represents a critical early event in the malignant transformation of these tumors. Indeed, a recent study showed that 11p15.5 alterations frequently occur in both embryonal and alveolar rhabdomyosarcoma [40]. Developing strategies for the early detection of 11p15.5 alterations and for targeting premalignant cells may provide a promising avenue for preventing these pediatric malignancies.

The 11p15.5 alterations are also associated with chemotherapy response, increasing in frequency from 50% before treatment, to 82% after chemotherapy, and 94% in recurrent tumors [22]. This suggests that either pre-existing 11p15.5-altered clones are more chemoresistant and thus undergo advantageous selection during treatment, or tumor cells acquire 11p15.5 alterations as an adaptive mechanism for survival under chemotherapy pressure.

**Fig. 2:**
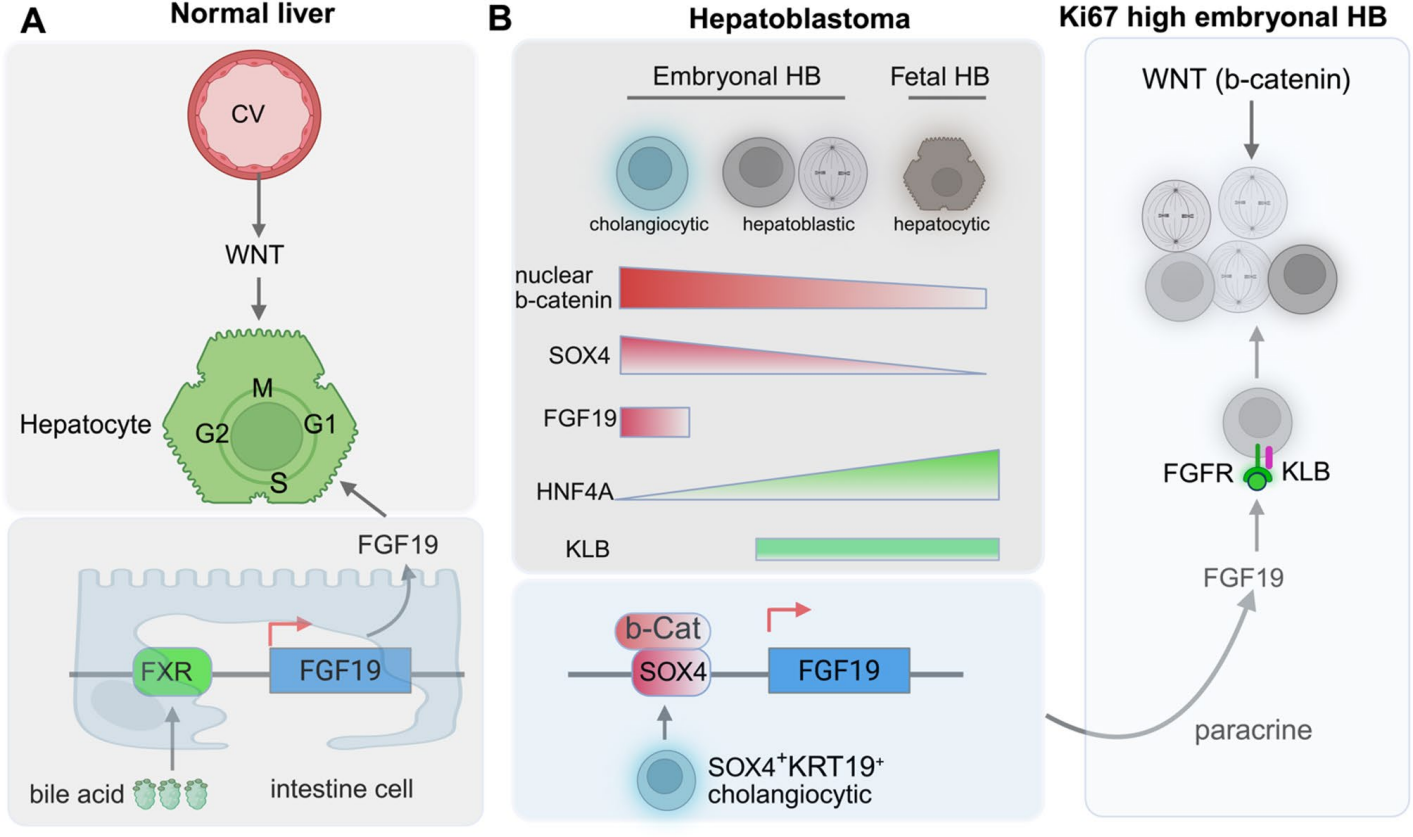
A biliary developmental program confers growth advantage to embryonal hepatoblastoma via FGF19 expression in a paracrine manner. (**A**) In normal liver, hepatocyte proliferation is stimulated by two factors: WNT from central vein (CV) endothelial cells promotes hepatocyte mitosis, and FGF19 from intestine and gallbladder epithelial cells promotes G1 to S phase transition. The expression of FGF19 (or its paralog Fgf15 in mouse) is regulated by the bile acid receptor FXR. (**B**) The scRNA-seq and spatial transcriptomics captured a unique population of cholangiocytic cells in embryonal hepatoblastoma (HB) characterized by high-WNT/b-catenin (b-Cat) signaling and high expression of SOX4, KRT19, and FGF19. FGF19 acts as a paracrine growth factor to promote cell proliferation of embryonal hepatoblastoma cells (high Ki67) expressing FGF19 receptor (KLB/FGFR1/4) with intermediate levels of WNT/b-catenin signaling. Distinct from its expression in normal liver, FGF19 expression is regulated by SOX4 and b-catenin in cholangiocytic cells

**Fig. 3:**
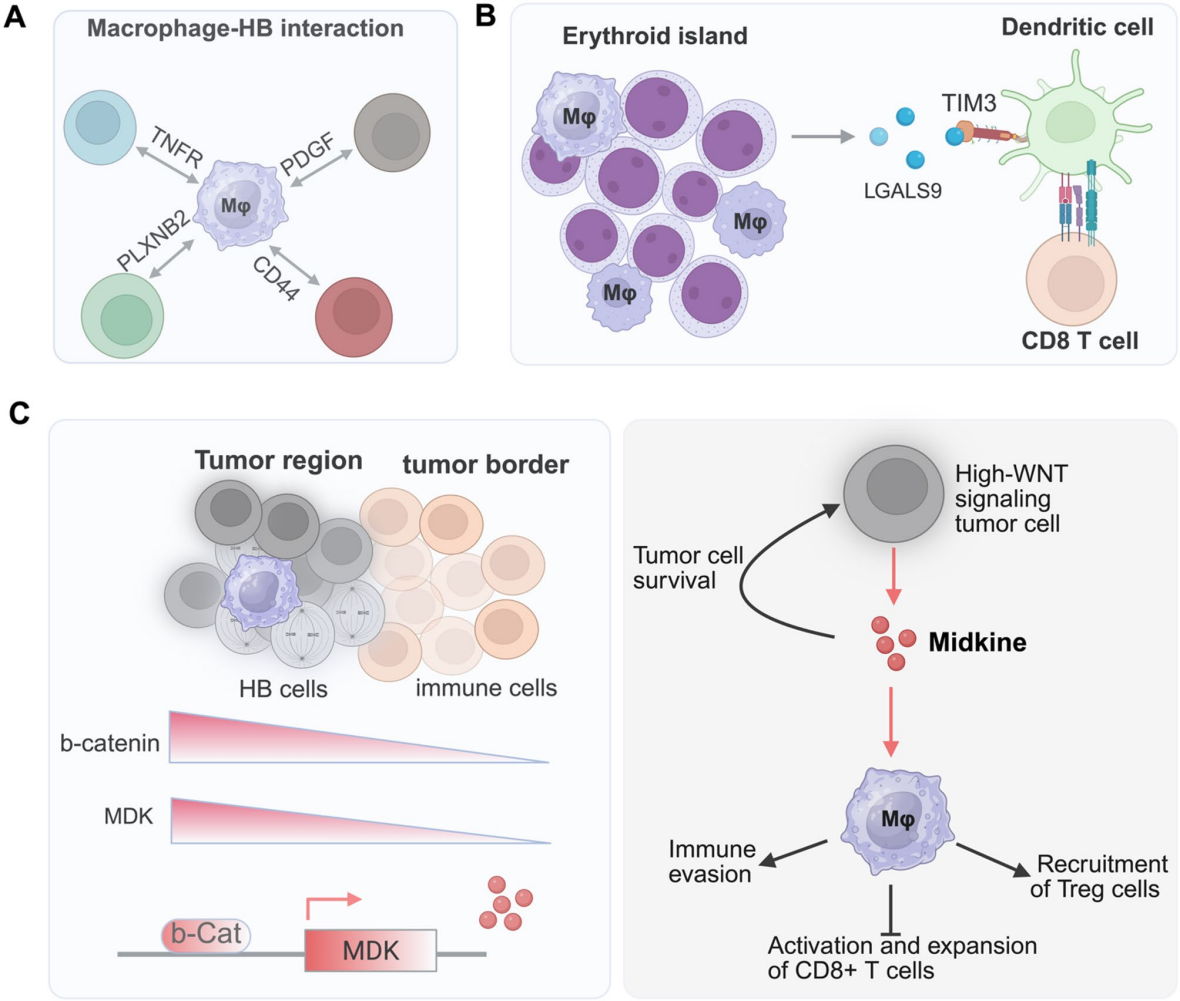
Mechanisms of tumor immune suppression in hepatoblastoma. (**A**) Song et al. [46] showed that MARCO^low^ macrophages (Mφ) in post-chemotherapy hepatoblastomas can interact with a heterogenous hepatoblastoma cell population through distinct signaling pathways involving CD44, PDGF, TNFR, and PLXNB2. (**B**) Wang et al. [56] showed that the erythroblastic islands consisting of VAMP1^+^ macrophages and erythroid cells may suppress immunity by interacting with erythroid cells and dendritic cells, which express ligand LGALS9 and receptor TIM3, respectively. (**C**) Munter et al. [48] showed that immune cells are excluded from hepatoblastoma (HB) tumor areas. The high WNT/b-catenin (b-Cat) signaling regulates the expression of MDK (midkine), which alters the phenotype of macrophages that mediate immune suppression

**Fig. 4:**
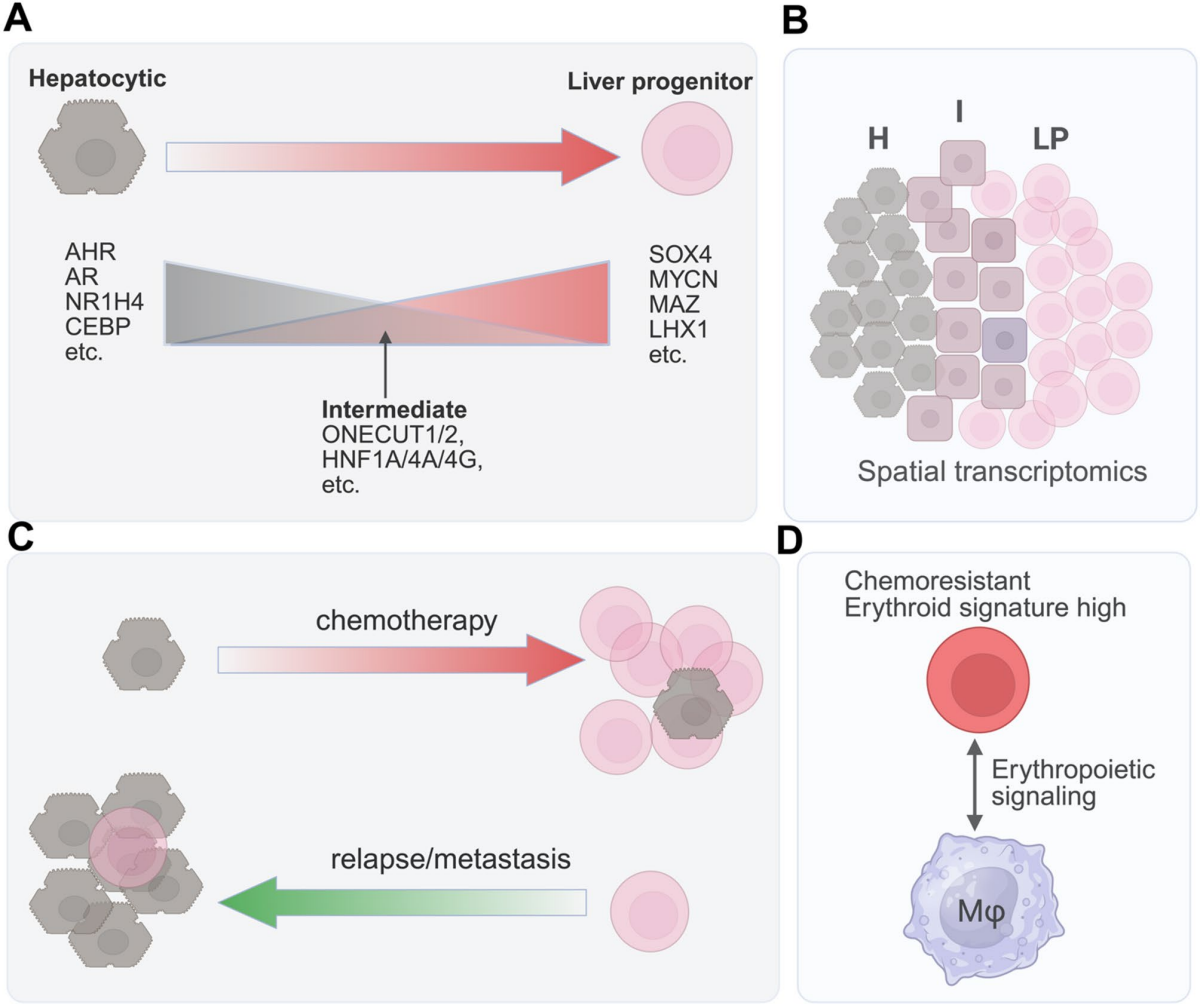
Cellular plasticity of hepatoblastoma during chemotherapy. (**A**-**C**) Roehrig et al. [49] showed that hepatoblastoma exhibits a continuum of cell states, spanning hepatocytic (H), liver progenitor (LP), and mesenchymal differentiation, with an intermediate (I) border between the H and LP populations in spatial maps. Chromatin-accessibility analysis showed distinct gene regulatory networks governing each differentiation state and the underlying cell state transitions. (**D**) Song et al. [46] identified a subgroup of hepatoblastoma cells that are chemoresistant, express high levels of erythroid genes, and specifically interact with macrophages (Mφ) through erythropoietic signaling

### Biliary developmental program cooperates with WNT signaling to drive the proliferation of hepatoblastoma

Despite its genetic simplicity, hepatoblastoma is a heterogeneous disease with distinct histologic and transcriptomic subtypes. The two major histologic types of hepatoblastoma are an epithelial type and a mixed epithelial and mesenchymal type. The epithelial type includes fetal and embryonal cell subtypes that often co-exist within the same tumor. Fetal hepatoblastoma cells resemble normal fetal hepatocytes. These cells are typically well-differentiated with low mitotic activity and are associated with better prognosis. Clinically, hepatoblastomas that meet the criteria for resection at diagnosis and exhibit well-differentiated fetal histology can be treated with surgery alone [41, 42]. In contrast, embryonal hepatoblastoma cells are less differentiated, have higher mitotic activity, and are associated with poorer prognosis. The mixed epithelial and mesenchymal type contains not only epithelial components but also mesenchymal elements, such as osteoid, cartilage, and fibrous tissue. However, the mechanism that determines the heterogeneity and differentiation state of hepatoblastoma cells is not well understood.

Previous studies using microarray and bulk RNA-seq approaches classified hepatoblastoma into distinct molecular subgroups, including C1 and C2 [18], C1/C2A/C2B [43], MRS-1/MRS-2/MRS-3 [23], hepatocytic/proliferative/mesenchymal [7], or hepatocytic/liver progenitor/mesenchymal subgroups [22]which are associated with different tumor histology and outcomes. Recent scRNA-seq, multi-omics, and spatial transcriptomics studies further revealed intratumoral heterogeneity of hepatoblastoma at the single-cell and spatial levels [44–49]. These studies emphasize that hepatoblastomas include a continuum of cell-differentiation states, between multipotent hepatobiliary progenitors and hepatocytic/cholangiocytic/mesenchymal cells, and clonal evolution with an immunosuppressive microenvironment. Despite these advances in understanding the heterogeneity of hepatoblastoma, a fundamental question remains––Why does universally activated WNT/b-catenin signaling drive hepatoblastoma into such diverse cellular and molecular states?

Using scRNA-seq combined with spatial transcriptomics, Wu et al. [45] provided new insight into how WNT/β-catenin signaling influences the differential proliferation rates of fetal and embryonal hepatoblastoma cells. Hepatocyte proliferation in the normal liver is driven by two distinct signaling pathways: First, bile acid stimulates farnesoid X receptors (FXRs) in ileal enterocytes to promote the increased expression of the FGF19 growth factor. FGF19, which is produced by ileal enterocytes and gallbladder epithelial cells, enters the portal circulation and, in turn, promotes G1/S-phase cell cycle transition in hepatocytes (Fig. 2A). Second, WNT released from the endothelial cells of the liver central vein promotes hepatocyte mitosis by inhibiting E2F7/E2F8 via the WNT target TBX3 [50–52]. However, Wu et al. demonstrated that in embryonal hepatoblastoma, FGF19 is produced directly from a small and unique population of hepatoblastoma cells that express a developmental biliary-lineage program and promote proliferation of embryonal hepatoblastoma cells in a paracrine manner. This paracrine FGF19 signaling acts in concert with constitutive activation of the WNT-signaling pathway, driven by gain-of-function mutations in *CTNNB1* or loss-of-function mutations in the tumor suppressor *APC* (Fig. 2B). One of these WNT-signaling pathway alterations are found in nearly all hepatoblastomas.

FGF19-producing cholangiocytic-like hepatoblastoma cells are characterized by high WNT/b-catenin signaling, a low proliferation rate, and expression of cholangiocyte markers *KRT19* and *SOX4*, a transcription factor important in biliary-lineage differentiation. These cholangiocyte-like cells are surrounded by embryonal hepatoblastoma cells, characterized by intermediate levels of WNT/b-catenin signaling, a high proliferation index (Ki67), and high expression of KLB, a hepatocyte transmembrane protein that plays a crucial role as a co-receptor for FGF19. Using tumoroids, Wu et al. [45] further validated that exogeneous FGF19 is important for the proliferation of KLB^+^ hepatoblastoma cells that do not express FGF19. FGF19-expressing cholangiocytic tumor cells have a selective growth advantage over hepatocytic tumor cells over multiple passages in a culture system. Interestingly, they found that the expression of FGF19 in the cholangiocytic-like cells is regulated directly by SOX4 and WNT/b-catenin and is decoupled from regulation by FXR. This study highlights that hepatoblastoma may originate from a multipotent hepatobiliary progenitor and that activation of a developmental biliary-lineage program can influence tumor evolution and heterogeneity by conferring a lineage-specific growth advantage. It also suggests that targeting the FGF19 receptor (FGFR) by small-molecular inhibitors currently in clinical trials may have antitumor effects on embryonal hepatoblastoma. Notably, FGF19 is amplified in a small percentage of hepatoblastomas [22].

### Fetal and WNT-high embryonal tumors exhibit distinct drug responses

Similar to Wu et al.’s study [45]Kluiver et al. [53] identified distinct WNT-signaling states in hepatoblastoma tumors and organoids by using multi-omics and spatial transcriptomics. They identified two distinct tumor epithelial signatures, a hepatic “fetal” type and a WNT-high “embryonal” type, that displayed divergent WNT-signaling patterns. The fetal type is enriched for liver-specific WNT targets (e.g., *GLUL*,* CYP2E1*,* RHBG*), whereas the embryonal type is enriched in canonical WNT-target genes (e.g., *AXIN2*,* NOTUM*,* NKD1*). Notably, the dichotomous expression patterns of the transcription factors HNF4A and LEF1 (a WNT-pathway transcription factor that interacts with b-catenin) clearly distinguish fetal from embryonal tumor cells.

Using a high-throughput drug screening of patient-derived tumor organoids, Kluiver et al. found that the embryonal tumor organoids and fetal tumor organoids are highly and equally sensitive to HDAC2 inhibitors, which is consistent with findings from a recent study by Espinoza et al. [54]. Interestingly, hepatoblastoma is more sensitive to the HDAC inhibitor romidepsin than are other embryonic tumors, such as Wilms tumor and neuroblastoma [53]. Romidepsin has been approved for treating cutaneous T-cell lymphoma and peripheral T-cell lymphoma. However, fetal tumor and embryonal tumor organoids exhibited differential sensitivity to receptor tyrosine kinase inhibitors: embryonal organoids were sensitive to FGFR inhibitors, but fetal organoids responded to EGFR inhibitors. They found that FGFR1 is highly expressed in WNT-high hepatic embryonal tumors, and FGFR4 and EGFR are highly expressed in HNF4A^+^ hepatic fetal tumors [53]. These findings suggest that FGF and EGF promote proliferation in both tumor cell populations. Withdrawal of EGF from culture medium significantly reduced fetal organoid growth but did not affect that of the embryonal organoids. Removal of FGF10 from culture medium had no effect on the growth of either organoid, leading Kluiver et al. to hypothesize that FGF signaling in these organoids is autocrine.

Wu et al. also noticed that some hepatoblastoma organoids make FGF19 for cell proliferation when EGF10 is withdrawn [45]. However, this is not mutually exclusive to the paracrine functions of FGF19. One early study showed that FGF19 can bind FGFR1 and FGFR4 with similar affinity in the presence of co-receptor KLB [55]. However, whether WNT signaling determines the expression of FGFR isoforms in fetal and embryonal tumors and that of EGFR in fetal tumors await further study.

### Immunosuppressive microenvironment of hepatoblastoma

Hepatoblastomas with liver progenitor cell features are immune “cold” [22]which means that compared with the well-differentiated hepatocytic-type hepatoblastoma, the liver progenitor–type samples are void of immune cells [49]. Using scRNA-seq analysis of post-chemotherapy tumors, Song et al. [46] investigated the relations between the TME and tumor heterogeneity. They found three immune cell populations, macrophages, pro-myelocytes, and basophils, enriched within the tumors. Subset analysis revealed that tumor-associated macrophages are the only cell type that exhibits transcriptomic differences, compared to the nontumor counterparts, characterized by attenuated expression of the scavenger receptor *MARCO* (*MARCO*^*low*^) in tumor macrophages. Predictions about ligand–receptor interactions suggested that these *MARCO*^low^ tumor–associated macrophages engage with different tumor populations through distinct signaling pathways [46] (Fig. 3A). Another scRNA-seq study analyzing treatment-naïve hepatoblastoma samples revealed an immune landscape marked by the aberrant accumulation of erythroblastic islands, composed of *VCAM1*⁺ macrophages and erythroid cells, which was inversely correlated with patient survival [56]. Erythroid cells suppress dendritic cell function through the LGALS9/TIM3-signaling axis (Fig. 3B), which might in turn lead to the impaired activation of antitumor T-cell responses [56].

Extramedullary hematopoiesis is a typical pathologic feature of hepatoblastoma, which may reflect an early developmental stage of hematopoiesis in liver due to a unique TME. While it remains unclear whether the *MARCO*^low^ and *VCAM1*^+^ macrophages represent the same population, Song et al.’s study identified a substantial erythroid cell population within hepatoblastoma. The enrichment of erythroid cells was consistent with findings from our genetic model, analyzed using scRNA-seq and spatial transcriptomics analysis [39]. These studies indicate that erythroid cells in an extramedullary hematopoiesis island may play an important role in promoting tumorigenesis.

Münter et al. [48] extended the paradigm by conducting single-cell and spatial transcriptomic analyses to uncover a continuous differentiation spectrum within hepatoblastoma with varying degrees of WNT-signaling activation (Fig. 3C). Strikingly, high WNT activity was associated with elevated expression of MDK (midkine), an immunomodulatory factor linked to immune suppression and evasion [57–60]. Compared to nontumor and fetal liver samples, hepatoblastoma tumors exhibited a marked reduction in B-cell infiltration and a decreased ratio of cytotoxic lymphocytes to regulatory T cells. Furthermore, B-cell abundance showed a negative trend with disease progression, including relapse and metastasis, compared to that in primary tumors. Spatial transcriptomics and immunofluorescence staining validated the exclusion of immune cells, particularly lymphoid cells, from tumor cell regions [48]. Immune evasion in hepatoblastoma was observed in both treated and untreated tumor samples. Further analysis revealed that macrophages within tumor regions express genes associated with immune suppression. Using an in vitro co-culture system, the study demonstrated that hepatoblastoma cells can induce a pro-fibrotic shift in macrophages, potentially mediated by MDK. Ligand–receptor interaction predictions suggested that MDK signals through receptors, such as NCL and LRP1, both of which are linked to immunosuppressive pathways [57, 61, 62]. Münter et al. further showed that MDK expression is regulated by b-catenin [48]. As a growth factor, MDK may also promote the survival, proliferation, and metastasis of hepatoblastoma cells (Fig. 3C), which has been reported in other types of cancers [63]. This study uncovered a WNT–MDK axis that drives immune evasion in poorly differentiated hepatoblastoma, thereby providing a mechanistic basis for the immune-cold phenotype observed in aggressive tumors and highlighting MDK as a promising therapeutic target to overcome immune resistance.

### Cell plasticity and chemotherapy resistance in hepatoblastoma

The current standard of care for hepatoblastoma includes surgical resection and systemic chemotherapy. Complete surgical removal of the tumor remains the strongest determinant of long-term survival. For unresectable tumors at diagnosis, neoadjuvant chemotherapy is used to shrink tumors, followed by surgical resection. Cisplatin is the cornerstone of chemotherapy regimens that have significantly improved outcomes of patients with hepatoblastoma. Doxorubicin is often added to cisplatin for the treatment of high-risk hepatoblastoma [64]. However, heterogeneous responses to chemotherapy and high relapse rates in aggressive subtypes pose substantial clinical challenges. Furthermore, acute toxicities and late effects of cisplatin are extremely common: 25–90% of patients with hepatoblastoma experience cisplatin-related ototoxicity, which affects language and social development [65]. Cisplatin is also associated with acute and chronic renal toxicity. Doxorubicin, in contrast, is associated with cardiomyopathy and development of secondary leukemia in hepatoblastoma patients [66].

Recent genomics, single-cell, and multi-omics studies have begun to uncover the molecular underpinnings of chemotherapy resistance [22, 46, 49]which might offer new opportunities to develop strategies for overcoming chemoresistance, improving patient survival, and perhaps limiting chronic toxicities of therapy by allowing for lower cisplatin cumulative doses. Hirsch et al. [14] utilized an integrative approach, including genomics and bulk-RNA sequencing, to show that the liver-progenitor subtype (correlating with embryonal histology) is enriched in cisplatin-resistant tumors. During chemotherapy, liver-progenitor cells accumulate massive loads of cisplatin-induced mutations with a specific mutational signature, leading to the development of heavily mutated relapses and metastases. These cells expressed high levels of stem cell markers (e.g., *AFP*,* MYCN*,* LIN28B*,* EPCAM*). The biliary marker *KRT19* is also highly expressed, indicating that these cells may exhibit features of hepatobiliary common progenitors in liver development. Longitudinal analysis revealed that the resistant clones were derived from a single common ancestor cell. In their following single-cell multi-omics and spatial transcriptomics study [49]Roehrig et al. identified a continuum of hepatoblastoma cell states, from hepatocytic, to liver progenitor, and mesenchymal differentiation, with an intermediate between hepatocytic and liver progenitor populations (Fig. 4A, B).

Chromatin accessibility landscapes revealed that distinct cell states in hepatoblastoma are governed by specific transcription factor networks. For example, liver progenitor cells exhibit high expression and transcriptional activity of SOX4 and MYCN, which gradually decrease along the differentiation axis toward intermediate and hepatocytic cells. In contrast, hepatocytic cells show elevated expression and transcriptional activity of AHR, NR1H4, and CPEBB/D, which progressively diminish toward less differentiated states. However, the intermediate cell state is enriched with transcriptional activity of HNF1A, HNF4A, and ONECUT1 that are involved in hepatocyte differentiation. Interestingly, a recent study showed that ONECUT1 is a tumor suppressor that inhibits YAP/β-catenin–driven hepatoblastoma formation in mice [67]. Notably, liver progenitor subclones, characterized by overexpression of stemness and DNA-repair genes, proliferate more rapidly after neoadjuvant chemotherapy. However, most tumor cells in some metastatic lesions displayed a hepatocytic phenotype (Fig. 4C). This study suggests that hepatoblastoma cell states are dynamic rather than static and are influenced by both genetic alterations and microenvironmental cues. These findings indicate that clonal evolution under therapeutic pressure selects for plastic, chemoresistant phenotypes capable of adapting to cytotoxic stress.

Song et al. identified not only a population of tumor-associated clusters expressing erythroid genes but also a subgroup of hepatoblastoma cells with an intrinsic erythroid gene signature. These tumor cells specifically interact with macrophages through erythropoiesis signaling [46] (Fig. 4D). When maintained in culture as organoids, the cells were broadly resistant to all tested drugs in the study. However, the biological characteristics of the cells remain elusive. Contamination of red blood cells during scRNA-seq preparation could be a potential concern; however, the persistence of the erythroid gene signature in organoid cultures argues against this explanation. Similarly, in our MYC-driven mouse model of hepatoblastoma, we identified a population of tumor cells with high expression of erythroid genes [39]. Cell lines derived from these tumors also maintained hemoglobin gene expression, as confirmed by RNA-seq analysis (unpublished data). Notably, tumors from this MYC-driven model exhibited resistance to doxorubicin treatment. These findings suggest that erythroid gene expression in hepatoblastoma cells is associated with intrinsic drug resistance and warrants further investigation.

However, caution should be taken when interpreting data from these recent multi-omics studies, as they do not provide direct evidence that the reported associations are causative of chemoresistance. Future studies with direct functional validation are needed to confirm these findings.

### Therapeutic vulnerability of hepatoblastoma

Hepatoblastoma is a very rare cancer (1.76 in 1 million) [68]. Although patient-derived xenograft models have been developed in recent years [69, 70]the number of established human hepatoblastoma cell lines remained limited until recently. Notably, XenTech have developed up to seven PDX-derived hepatoblastoma cell lines, which have been employed in multiple preclinicalstudies [70–75]. These models have significantly expanded the toolkit available for hepatoblastoma research and have played a key role in advancing translational efforts, including drug screening and mechanistic studies. However, genetically engineered mouse models that recapitulate human disease are still rare, which has substantially hindered preclinical testing of novel therapies, particularly immunotherapies. As a result, few therapies are specifically targeted against hepatoblastoma, and most clinical trials rely on basket designs that include diverse pediatric solid tumors. Currently, there are 10 active early phase clinical trials targeting relapsed and refractory pediatric solid cancers, including hepatoblastoma (Table 1). These include two chemotherapy-based regimens, five immunotherapies, and three targeted therapies. Among the targeted approaches, tegavivint disrupts the interaction between β-catenin and TBL1, promoting degradation of β-catenin, the key oncogenic driver in hepatoblastoma [76]. Glypican-3 (GPC3), which is highly expressed in both hepatoblastoma and hepatocellular carcinoma, has emerged as a promising immunotherapeutic target. A recent clinical study in adults with liver cancer demonstrated that GPC3-directed CAR T cells co-expressing IL-15, but not GPC3 CAR T cells alone, achieved a disease control rate of 66.7% (8 of 12 patients) and an objective response rate of 33.3% (4 of 12 patients) [77]. These findings suggest that IL-15–armored GPC3 CAR T cells is a promising therapeutic strategy for patients with recurrent hepatoblastoma.

**Table 1:** Clinical trials for pediatric and young adult patients with relapsed or refractory cancer, including hepatoblastoma

Clinical Trials.gov Identifier (NCT)	Clinical Trial Phase	Treatment Modality	Therapy	Mechanism(s) of Action
04337177	1	Chemo	Flavored, oral irinotecan (VAL-413) with temozolomide	Combined chemotherapy
04901702	1	Chemo	Onivyde with talazoparib or temozolomide	Combined chemotherapy
04897321	1	Immuno	CAR T cells	B7–H3
04928677	1/2	Immuno	Codrituzumab	GPC3
06521567	1/2	Immuno	Cobolimab plus dostarlimab	Antibodies against TIM and PD1
046334357	1/2	Immuno	ET1402023 T cells	TCR mimic antibody to target AFP-peptide/HLA-A2 complex
03618381	1	Immuno	CAR T cells	EGFR
04851119	1/2	Targeted	Tegavivint	β-catenin
04308330	1	Targeted	Vorinostat with chemotherapy	HDACi with chemotherapy
02867592	2	Targeted	Cabozantinib-S-malate	Tyrosine receptor kinases (VEGFR2, MET, RET)

Abbreviations: AFP, α-fetoprotein; CAR, chimeric antigen receptor; Chemo, chemotherapy; EGFR, epidermal growth factor receptor; GPC3, Glypican-3; HDACi, histone deacetylase inhibitor; HLA, human leukocyte antigen; Immuno, immunotherapy; NCT, national clinical trial; PD1, programmed cell death protein1; TCR, T-cell receptor repertoire; TIM, T-cell immunoglobin

Due to the scarcity of disease models and limited functional studies, our understanding of cancer dependencies in hepatoblastoma remains limited, hindering the development of more effective and less toxic targeted therapies. Genome-wide CRISPR screenings, such as the Cancer Dependency Map (DepMap) Project at Broad Institute have provided valuable insights into cancer-dependency genes across many tumor types. The DepMap portal includes only two hepatoblastoma cell lines, Huh6 and HepG2. After excluding common essential genes, analysis of these two cell lines revealed only 210 shared cancer-dependency genes (Supplementary Table 1). The hepatoblastoma driver genes *CTNNB1* and *NFE2L2*, along with liver-specific transcription factors *HNF1A* and *HNF4A*, were also found to be essential for cell survival. These hepatoblastoma-dependency genes are particularly enriched in pathways related to mitochondrial translation and oxidative phosphorylation (Supplementary Table 1), suggesting that hepatoblastoma cells are especially vulnerable to disruptions in mitochondrial function. However, our recent CRISPR-screening study demonstrated that cancer cells exhibit markedly different behaviors under normoxic 2D culture conditions (as used in DepMap), compared to those under hypoxic 2D or normoxic 3D culture conditions [78]. These differences are particularly pronounced in genes associated with mitochondrial processes, calling into question whether targeting mitochondrial function would be effective in more physiologically relevant settings. Unfortunately, the overall number of targetable dependency genes identified from the two hepatoblastoma cell lines is low (Table 2), and most have not yet been functionally validated in relevant disease models.

**Table 2:** Targetable dependency genes identified in hepatoblastoma cell lines

Target	Description of Target	Target Inhibitor(s)
ABHD17A	Anhydrolase domain containing 17 A, depalmitoylase	ABD957
ACLY	ATP citrate lyase	Bempedoic acid
CTNNB1	Β-catenin	Tegavivint, foscenvivint
CCND1	Cyclin D1	Palbociclib, ribociclib, abemaciclib
EP300	E1A-binding protein P300	CPI-637, NEO2734
Complex 1	NADH: ubiquinone oxidoreductase	IACS-010759, GBS-01, ASP4132, EVT-701
GGT1	Gamma-glutamyl transferase 1	OU749
IGF1R	Insulin-like growth factor 1 receptor	Xentuzumab, AXL-1717, linsitinib
KAT2A	Lysine acetyltransferase 2 A	GSK-4027, GSK-983, AUTX703
MAPK1	Mitogen-activated protein kinase 1	Ulixertinib, LY3214996, ravoxertinib, CC-90,003
MVD	Mevalonate diphosphate decarboxylase	Statins
PC	Pyruvate carboxylase	ZY-444
PI4KB	Phosphatidylinositol 4–kinase beta	PI-273, PIK93
PIK3C3	Phosphatidylinositol 3–kinase catalytic subunit type 3	SAR405
PTK2	Protein tyrosine kinase 2	Defactinib, BI-4464, PF-431,396
USP9X	Ubiquitin-specific peptidase 9X-linked	FT709

### Future directions

Hepatoblastoma is characterized by intratumoral heterogeneity in WNT signaling, clonal evolution, and both epigenetic and transcriptional plasticity, all of which may contribute to the development of chemoresistance. Despite these insights, the mechanisms underlying the distinct WNT-signaling activity associated with therapy response remain poorly understood. Activating mutations in *CTNNB1* can either arise through missense mutations, which are more common in younger patients (< 4 years), or through exon 3 deletions, which tend to occur later in life [22]. The point mutations in *CTNNB1* predominantly occur in patients with locus 11p15.5 mosaicism [26].

Mouse models of hepatoblastoma have demonstrated that *CTNNB1* point mutations, in combination with the *YAP1* oncogene, result in a more differentiated tumor phenotype than that of *CTNNB1* exon 3–deletion mutant [79]. A deeper understanding of how WNT-pathway diversity intersects with therapy-resistant progenitor cell populations may reveal novel therapeutic strategies to overcome resistance and prevent disease relapse. Although immunotherapies, such as anti-GD2 therapy, have substantially improved outcomes in neuroblastoma, effective immunotherapies for hepatoblastoma remain underdeveloped. Targeting the immune-cold nature of liver progenitor tumors may be key to eradicating recurrent and metastatic disease. Despite recent advances in understanding the complex biology of hepatoblastoma, chemotherapy remains the cornerstone treatment for high-risk disease.

Improving the efficacy of chemotherapeutic agents while minimizing toxicity is a pressing clinical need. In our recent genome-wide CRISPR screening, we identified *PRKDC* as a genetic modifier of doxorubicin response. Inhibition of PRKDC enhanced the efficacy of doxorubicin in both genetically engineered mouse models and patient-derived hepatoblastoma xenografts [39]suggesting that identifying genetic modulators of chemotherapy response is a powerful strategy to optimize current treatment regimens.

Many recent multi-omics studies in hepatoblastoma are based on limited sample sizes and experimental models that may not fully capture the complexity of human disease. While these studies provide valuable mechanistic insights, further validation in larger, well-annotated patient cohorts is needed to confirm their relevance to tumor evolution and chemoresistance.

Another challenge is the lack of a unified definition for the cell types identified in recent single-cell and spatial transcriptomic studies of hepatoblastoma. Variability in technological platforms, analytical methods, limited sample sizes, and differences in treatment status (pre- vs. post-chemotherapy) have contributed to inconsistent characterization of tumor subpopulations. The lack of standardization in nomenclature of cell populations hinders efforts to fully understand hepatoblastoma heterogeneity and to define reproducible, clinically relevant tumor cell states. Thus, more robust analytic tools may need to be developed, and all datasets may need to be integrated for a comprehensive analysis of hepatoblastoma samples.

## Electronic supplementary material

Below is the link to the electronic supplementary material.

Supplementary Material 1

Supplementary Material 2

## Data Availability

No datasets were generated or analysed during the current study.
