# Supplementary material for: From preneoplastic lesion to heterogenous tumor: recent insights into hepatoblastoma biology and therapeutic opportunities
Source: Mol Cancer. 2025 Jul 19;24:198. doi: 10.1186/s12943-025-02405-8 (PMC12275271; doi:10.1186/s12943-025-02405-8)
Supplement: Supplementary file 2 — Supplementary Material 2 [file 12943_2025_2405_MOESM2_ESM.docx]

**Supplementary Figure 1. *IGF2* expression across different cancer cell types.** Data were extracted from the DepMap portal ([www.depmap.org](http://www.depmap.org)).
